# Supplementary material for: Improving Bone Health in Patients with Metastatic Prostate Cancer with the Use of Algorithm-Based Clinical Practice Tool
Source: Geriatrics (Basel). 2022 Nov 24;7(6):133. doi: 10.3390/geriatrics7060133 (PMC9778212; doi:10.3390/geriatrics7060133)
Supplement: Supplementary file 1 [file geriatrics-07-00133-s001.zip › geriatrics-1962140-supplementary.pdf]

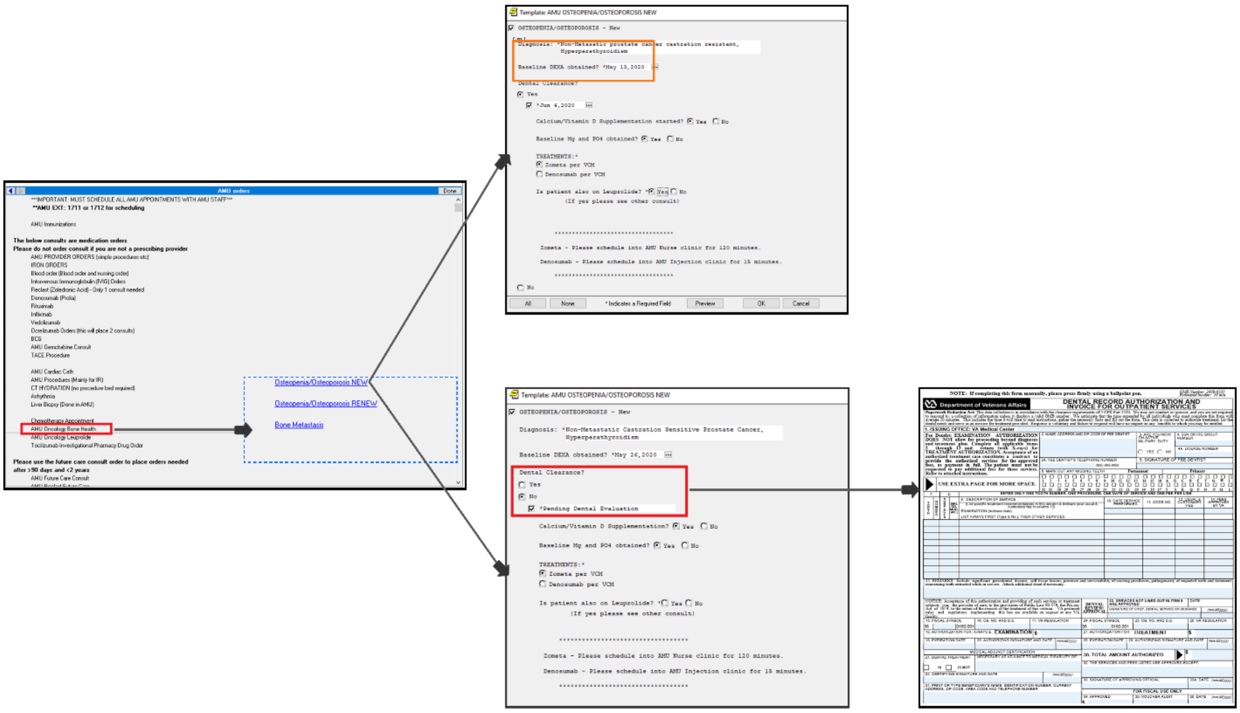

Supp. Figure S1. Algorithm explaining the steps while prescribing BMA in case of prostate cancer with new diagnosis of osteopenia/osteoporosis.

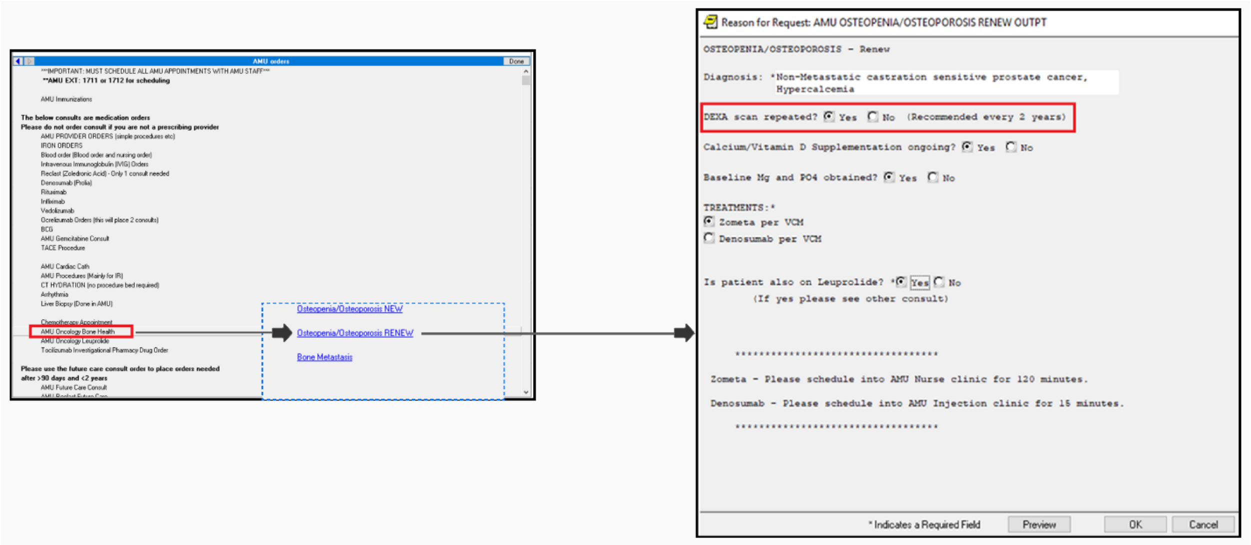

Supp. Figure S 2. Algorithm explaining the steps while renewing the prescription of BMA in case of prostate cancer with known diagnosis of osteopenia/osteoporosis.

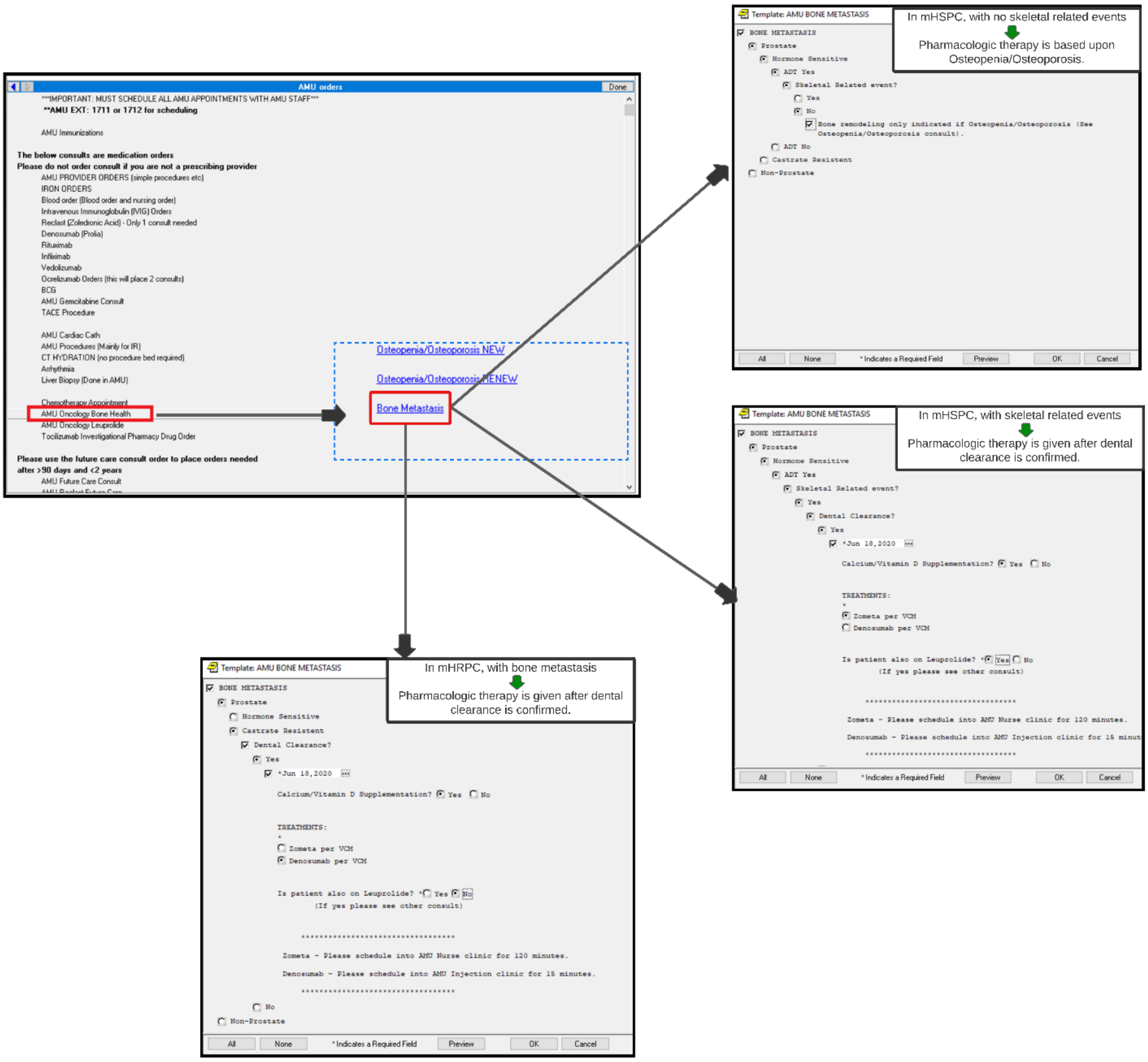

Supp. Figure S3. Algorithm explaining the steps while prescribing BMA in case of prostate cancer with bone metastasis.
